# A critical reflection on using the Patient Engagement In Research Scale (PEIRS) to evaluate patient and family partners' engagement in dementia research

**DOI:** 10.3389/frdem.2024.1422820

**Published:** 2024-06-24

**Authors:** Joey Wong, Lillian Hung, Cates Bayabay, Karen Lok Yi Wong, Annette Berndt, Jim Mann, Lily Wong, Lynn Jackson, Mario Gregorio

**Affiliations:** ^1^School of Nursing, University of British Columbia, Vancouver, BC, Canada; ^2^UBC IDEA Lab, School of Nursing, University of British Columbia, Vancouver, BC, Canada; ^3^School of Social Work, University of British Columbia, Vancouver, BC, Canada

**Keywords:** patient and public involvement, aging, dementia, older adults, technology, evaluation

## Abstract

**Introduction:**

Research involvement of people with lived experiences is increasing. Few tools are designed to evaluate their engagement in research. The Patient Engagement In Research Scale (PEIRS) is one of the few validated tools. Our team employed the PEIRS with patient and family partners with lived experiences of dementia every 6 months in a two-year telepresence robot project. This reflection paper reports our self-study on key learnings and proposes practical tips on using the PEIRS to evaluate patient and family partners' engagement in dementia research. It is the first to document a case using the PEIRS multiple times in a dementia research project.

**Methods:**

Guided by Rolfe et al.'s reflective model, we conducted three team reflective sessions to examine the team's experiences using the PEIRS to improve and evaluate patient and family partners' engagement in the research. We also reviewed our meeting notes and fieldnotes documented in the research journal. A reflexive thematic analysis was performed.

**Results:**

The team identified three key learnings: the values of using the PEIRS survey, the adaptations, and the factors influencing its implementation as an evaluation tool. Using the PEIRS provided significant benefits to the project, although some patient and family partners felt it was burdensome. The evaluation tool was enhanced with emojis and comment boxes based on suggestions from patient partners. The emojis introduced an element of fun, while the comment boxes allowed for personalized responses. Several factors influenced the PEIRS tool's effectiveness: the interviewer's identity, the confidentiality of responses and follow-ups, the timing and frequency of using the tool, and the presentation of the evaluations. These learnings led to the development of six practical tips,—“ENGAGE”: Enjoyable and fun process, Never impose, Get prepared early, Adapt to the team's needs, Give people options, and Engage and reflect.

**Conclusion:**

With the emerging trend of including people with lived experiences in dementia research, there is a need for ongoing assessment of engagement from both patient and family partners and the research team strategies. Future research can further explore survey logistics, co-development of evaluation tools, and the use of tools with people living with dementia.

## 1 Introduction

The involvement of people with lived experiences in health research has become increasingly important and continues to gain acceptance in the research field worldwide (L'Espérance et al., 2021). Many organizations and funding bodies now mandate the involvement of these individuals—referred to as “patient partners”—throughout various stages of research [Patient-Centered Outcomes Research Institute (PCORI), 2014]. Patient partners encompass patients, persons living with the disease, caregivers, family members, and friends [Patient-Centered Outcomes Research Institute (PCORI), 2014; Strategy for Patient-Oriented Research (SPOR), 2014]. Because of the increase in patient involvement in research, it is imperative to evaluate the quality of engagement of patient partners in the process.

While numerous existing frameworks support and evaluate patient and public involvement in research (Greenhalgh et al., 2019), there is a dearth of tools specifically designed to evaluate the quality of patient partner engagement in research (Boivin et al., 2018). The Patient Engagement In Research Scale (PEIRS) is a measurement tool to evaluate the quality of engagement of patient partners in research (Hamilton et al., 2018a,b). The original 37-item PEIRS evaluation tool was shortened and validated to the 22-item (PEIRS-22) questionnaire (Hamilton et al., 2021). The PEIRS-22 is organized across eight subthemes: *procedural requirements, convenience, contributions, support, team interaction, research environment, feel valued*, and *benefits*. In a recent systematic review of tools for assessing health research partnership outcomes and impacts, the PEIRS-22 scored high in scientific rigor and usability (Mrklas et al., 2023). Besides being a one-time measurement, the PEIRS-22 can support the research team in continuously improving patient engagement in the research project (Hamilton et al., 2021). The information from the PEIRS-22 evaluation allows researchers and patient partners to work together on diverse patient engagement strategies to improve engagement experiences. The PEIRS-22 can then serve as a tool to measure and capture any improvements over the research process after applying the strategies. The PEIRS-22 allows a “feedback loop” for progress monitoring and ongoing improvements in the research team (Hamilton et al., 2021).

The PEIRS-22 tool has been used in several research studies to evaluate patient partner engagement in people living with Parkinson's Disease (Morel et al., 2023) and Down Syndrome (Chung et al., 2021). The tool has also been used to foster inclusivity of underrepresented populations in adults with congenital heart disease (Messmer et al., 2023) and Parkinson's Disease (Sanchez et al., 2022). In some studies, the PEIRS-22 was employed to assess community stakeholder engagement (Barn et al., 2022; Morse et al., 2023). Moreover, the PEIRS-22 has also been translated, culturally adapted, and linguistically validated into Danish to assess patient partner engagement in cancer patients (Christiansen et al., 2023). In all these studies, the PEIRS-22 was a pragmatic tool for researchers to appraise patient partners' experiences and engagement throughout the research process.

In Canada, the PEIRS-22 is being employed in nationwide collaborative action research to develop a Canadian evaluation framework for patient and public engagement in research (L'Espérance et al., 2021). The Strategy for Patient-Oriented Research (SPOR) Evidence Alliance, a national, multilevel organization, has utilized the PEIRS-22 during a self-study “to reflect on the experiences of patient involvement in the organization's first 3 years” (Li et al., 2022, p. 30; Wang et al., 2023).

While a number of recent studies have utilized the PEIRS-22 in evaluating patient perspectives on meaningful engagement, only one recent commentary paper was found that planned to adopt the PEIRS tool to evaluate meaningful engagement in people with lived experience of dementia who were members of the Advisory Group for the Canadian Consortium of Neurodegeneration and Aging (CCNA) in dementia research (Snowball et al., 2022). Snowball et al. (2022) shared a plan to use the PEIRS-22, with two questions from the original PEIRS-37 and free text responses for a one-time evaluation at the end of the first year of their research project, evaluating the experiences of the Advisory Group members.

To enhance patient and public engagement in our patient-oriented study on implementing telepresence robots in long-term care, our team used the PEIRS-22 questionnaire to assess the experiences of patients and family members with lived experiences of dementia as partners during the two-year partnership in the Telepresence Robot project. In the study, telepresence robots, a tablet on wheels that allows virtual communication between family members and residents, were placed in residents' rooms in four Canadian long-term care homes. Family members could call in from around the world anytime and control the robot's movement in the resident's room. The project explores the experiences of residents, family members, and staff members who have adopted telepresence robots in long-term care. The results of the project were published in another papers (Hung et al., 2023; Ren et al., 2024). The research team included people living with dementia, family partners, frontline staff, community partners, researchers and trainees. The involvement of people living with dementia and family partners started from the planning stage of the research. One person living with dementia is the project co-lead. Patient and family partners were engaged in monthly team meetings via Zoom to discuss data collection, staff engagement strategies, data analysis, manuscript preparation and conference presentations.

Before using the PEIRS-22, the research team had an orientation session on an overview of the PEIRS-22 survey and a discussion on using the PEIRS-22 tool in the Telepresence Robot project. The team decided to digitize the PEIRS-22 survey. Patient and family partners suggested supplementing the numerical rating scale of the PEIRS-22 with emojis. A comment box was added to each question for contextual or additional information. The team also talked about the interview formats. After the discussion in the orientation session, the evaluation team digitized the questionnaire using an online software application, Qualtrics, added emojis to the 5-point Likert scale and comment boxes to each question for free text responses. The research team decided to perform evaluations using the PEIRS-22 questionnaire from the start of the project. It continued every 6 months for four sessions to evaluate engagement at different time points in the project. The intention was to identify gaps and make improvements throughout the project.

**Figure 1:**
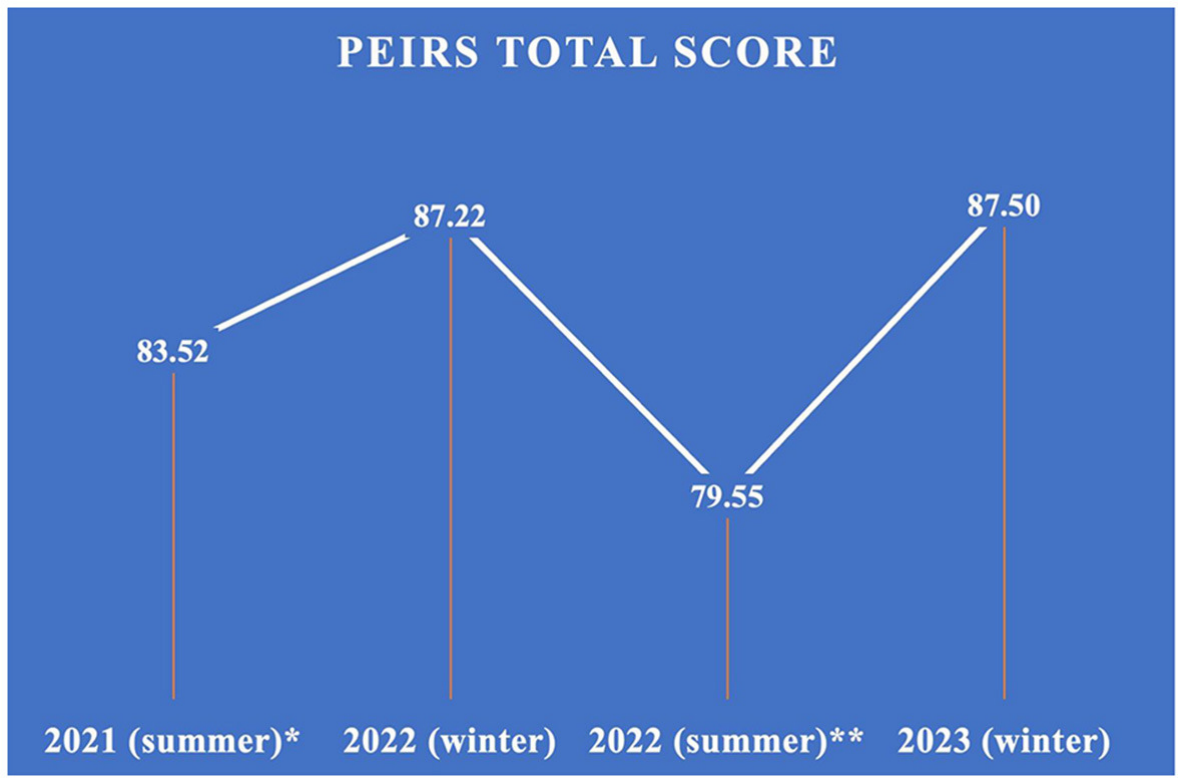
PEIRS total scores (*with 9 blank answers; **with 3 blank answers).

The first evaluation took place with a round of interviews in the summer of 2021. This round of interviews was one-to-one conversations with each patient and family partner via Zoom at a date and time convenient to the partner. Respecting partners' autonomy and choice, subsequent evaluations were done independently through the online survey by the partners, except for one family partner who preferred Zoom evaluations. There were, in total, four rounds of evaluations. Through the evaluations, our team learnt about what worked and what did not regarding the team's engagement performance. The research team adopted different engagement strategies to improve patient and family partner engagement in the project based on the feedback received in each round of evaluations, e.g., creating newsletters for sharing the project progress and how the robots were being used at each long-term care site, providing clear tasks information and task subgroups (e.g., manuscript writing and staff engagement strategies) for partners to choose to be involved in their preferred tasks. The average total scores of the four rounds of evaluations are shown in Figure 1. A higher score indicates a greater meaningful engagement (Christiansen et al., 2023). Overall, the patient and family partners remarked positively on their experience with the research team and the project work (see Table 1).

**Table 1:** Examples of comments from patient and family partners.

**PEIRS domains**	**Comments**
Procedural requirements	The partners praised the research team for the level of attention, engagement and communication: “Organizers are efficient and send organized information.” However, some partners voiced out that there needed to be an external source of communication besides team meetings as they might include too much information. The team moved some information in the email. After that, the team found a more innovative way than emailing, which was sharing information through our team newsletters. The team received encouraging comments: “Fabulous newsletter! Very engaging design and succinct messaging. As mentioned above, the newsletter really provided a great opportunity to get to know one another through photos and feature stories.”
Convenience	In the beginning, some partners voiced that “I did not have an opportunity to discuss my role in the project… participants should be asked if they feel that they are useful in the project.” Some partners found the research team's information concerning task assignments unclear. With this feedback, the research team created task subgroups and described the options of tasks that patient and family partners could join. Some partners found their involvement with the project convenient and manageable: “The team lead would always provide an option to talk over the phone at a time that was mutually convenient; also, we were welcome to email additional thoughts. The atmosphere was very friendly and forthcoming.” “The tasks don't take a lot of time. The tasks are very doable.” “Deadline flexibility is always appreciated.”
Contributions	The partners contributed their knowledge and perspectives and felt their contributions were well received: “The voices of participants are important, and mine was included.” “Being given the opportunity to share my lived-experience perspective in a constructive, forward-looking way is really why I continue to be part of this.”
Team environment	The partners felt there was trust and respectful partnership within the team: “The friendliness of the research team goes a long way to foster a good environment of trust.” “I'm treated with respect. We can say our view without judgement.” “Everybody is very respectful. They respect the silence; there is no expectation to have something to say all the time.”
Support	The partners felt well supported in their tasks and roles for the (Telepresence Robot) project: “Dr. Hamilton came in to explain the PEIRS (orientation), plus the newsletter and articles the team sends.” “Whether by email or by phone (or in-person), I was always able to reach the team leader.”
Feel valued	The partners felt their contributions were appreciated well and recognized through honoraria gifts, inclusion in events (e.g., conferences), and co-authorship in publications. “The Save-On and Amazon gift cards (vs. an honorarium check honorarium) are an excellent idea, although authorship is the gold standard of recognition.” “My name will be mentioned along with the author, and I get gift cards. I was invited to the Christmas party and the picnic (I appreciated that I was included in these).”
Benefits	The partners found their involvement beneficial to others as well as themselves. “It is a major and much-needed confidence boost! Personally, I think it really made a huge psychological difference going forward. Thanks so much!” “I feel it's a worthwhile project for community use.” “I always left the meetings with a sense of optimism and “can do” spirit.” “I believe it keeps me active, and it helps my cognition.” “Being involved in this project was also “therapeutic” for me in the sense that my experiences, both positive and negative, didn't just stay with me to be forgotten. Knowing that my lived experience and practical knowledge have a place to go with the potential to contribute to something positive in a field of healthcare that is often portrayed (and experienced) as negative offers hope for a better future. Society at large seems to falter in knowledge transmission, and these kinds of patient/family partnerships offer the opportunity to ensure that intergenerational, interprofessional, and other neglected interstitial connections are built up, maintained and can flourish.”

This study aims to reflect on key lessons learned and share practical strategies for using the PEIRS-22 in evaluating the engagement of patient partners living with dementia and family partners in research. It is the first to document a case of repeated application of PEIRS-22 within a dementia research project. The study will contribute to the growing science of patient and public engagement, particularly on meaningful engagement for people with lived experiences, using engagement evaluation tools, and advancing appraisal techniques to bolster public and patient engagement in dementia research.

## 2 Methods

The team performed the PEIRS-22 evaluation with patient and family partners every 6 months for four sessions until July 2023. Evaluators of the scale took reflective notes after interviews. The team then reflected on the experiences of using PEIRS in evaluating patient engagement in the Telepresence Robot project. There were three 1-h reflection sessions facilitated by JW via Zoom meetings. Team members who joined the reflection included project leads LH and JM (a patient partner co-lead), patient and family partners AB, LJ, LW and MG, project coordinator JW, evaluator team lead CB, and project team member KW. People living with dementia in our project are in the early stages of dementia. JM is living with Alzheimer's disease. LJ is living with frontotemporal dementia. MG is living with vascular dementia.

Rolfe et al. (2001) reflective model guided the reflection sessions. This model was chosen because it has been widely adopted for team reflection in healthcare research. It includes three main questions: What? (What is it?), So what? (Why is it important?), and Now what? (What should we do next?) We converted these questions into questions which fit our context: “What did we do well and not so well with the PEIRS-22 evaluation?” “What worked about the PEIRS-22 in dementia research, and what didn't work?” “Why did we/the PEIRS-22 do well or not so well?” “How can we do better in the future?” “How can the PEIRS-22 or other evaluation tools be improved in the future?” Reflection sessions were audio-taped and transcribed.

Following Braun and Clarke (2022) reflexive thematic analysis, we repeatedly read the reflective notes, which are transcriptions from our reflection sessions and listened to the recording to immerse ourselves in the data. We clustered related codes into categories and arranged these into themes. Reflective notes, transcripts, codes, categories, and themes were constantly compared to ensure consistency and coherence in the analysis. The data collection and analysis processes were iterative. Preliminary findings from the data of our prior reflection session informed the questions we asked in our next reflection session.

Trustworthiness refers to the fact that readers find the findings credible (Tracy, 2010). In other words, they can believe in the findings because the findings are based on comprehensive data sources and rigorous analysis and reflection processes. We enhanced the trustworthiness of our reflection by having more than one data source (reflection notes and transcripts) and practicing reflexivity with members from diverse backgrounds, challenging each other's assumptions in the reflection process.

### 2.1 Ethical considerations

Ethics approval was granted from the University Ethics Boards (H21-00844). Participants provided verbal consent and were offered the option to be identified by their actual names or pseudonyms in the dissemination of findings. Each participant has reviewed and approved the contents of this article.

## 3 Results

Telepresence Robot research team member characteristics are summarized in Table 2. Ten research team members are male, and 17 are female. The intergenerational team involves older adults and students. Patient and family partners made up about 25% of the research team. The rest of the team included two nurses, two recreation staff, one social worker, one community partner, six undergraduate and six graduate student trainees, and two academic professors. While all team members had some research experience in general, 11 had more experience in patient-oriented research.

**Table 2:** Telepresence robot research team member characteristics.

**Variable**	**Number (*n* = 27)**
**Disciplines**	
Patient partner living with dementia	3
Family partner	4
Nurse	2
Undergraduate student trainee	6
Graduate student trainee	6
Academic professors	2
Community partner	1
Recreation staff	2
Social worker	1
**Gender**	
Female	17
Male	10
**Research experience in general**	
Yes	27
**Experience in patient-oriented research**	
Yes	11

After the team critically reflected on using the PEIRS-22 to evaluate the engagement experiences of patient and family partners in our Telepresence Robot project, three key learnings were identified: the value of using the PEIRS survey, the adaptations, and the factors influencing its implementation as an evaluation tool.

### 3.1 The value of using the PEIRS survey

#### 3.1.1 The value of the project and beyond

When reflecting on the general impression of using the PEIRS-22, two members appreciated the inclusion of this evaluation tool in the project. The different subthemes of PEIRS offered a structured framework to evaluate and improve the research team's engagement progress in a holistic manner throughout the research process. Our patient partner, MG, provided his comments on having the PEIRS evaluation as the project progressed, “PEIRS is an excellent way of tracking our performance [on engaging patient and family partners] on a project, and I think it is a very good tool.” Our family partner, AB, also commented on the diverse aspects of engagement that PEIRS covered, “The questions do prompt you to draw your attention to things you might not have thought of otherwise.” The structured framework also helped evaluate and improve the research team's engagement of patient and family partners. From the field notes of the evaluations, under the subtheme “Convenience,” some partners commented at the beginning of the project on their roles in the research team: “I did not have an opportunity to discuss my role in the project… participants should be asked if they are feeling that they are useful in the project.” Some partners expressed that the research team's information concerning task assignments was unclear. The research team responded by creating task subgroups and providing information on different options of the tasks that patient and family partners could join. The research team also shared information on project subgroups and tasks in the monthly newsletters. The evaluations included the voices of patient and family partners, which helped the research team enhance the research engagement process.

Besides acknowledging the positive impact that the PEIRS-22 can bring to an individual project, our patient partner project co-lead, JM, added the values of adopting PEIRS-22 beyond the Telepresence Robot project, “there are learnings [from the PEIRS] at different points. Those learning can still contribute to the field. The research lab can take that input into [the engagement strategies of] future projects, on what works and what does not.” The PEIRS-22 provides a method to assess and compare patient and family involvement across research projects in our research lab and the dementia research field. Another patient-oriented project in our research lab adopted the PEIRS-22 after its use in the Telepresence Robot project. Another project lead, LH, stated, “it is not only for us [our research lab]. We can contribute to the field and promote the way we do patient-oriented research.”

#### 3.1.2 The value to patient and family partners and trainees

When asking members about how the PEIRS-22 helped individual members' engagement in the team, a family partner, AB, shared how the evaluations helped her reflect on her participation in project tasks and contributions to the research process:

“PEIRS is a tool that is helpful in some ways in terms of awareness. Raising awareness of my engagement […] You sort of realize, okay, this is where I spent my time and how I spent it. I think that is kind of a reflection. It's actually a pretty good thing. It makes me a little bit more aware of what I am or am not doing […] It certainly enhances our understanding of the various approaches to involvement in the project.”

The PEIRS-22 evaluations provided a platform for patient and family partners to share positive feedback and gaps/opportunities to improve the engagement experiences. These comments in the evaluation interviews might not often be shared in regular team meetings. The feedback encouraged and motivated student trainees in the research team to reflect, learn, improve, and use different strategies to engage patient and family partners meaningfully. For example, some comments created a positive team spirit: “I always left the meetings with a sense of optimism and “can do” spirit,” and “The tasks don't take a lot of time. The tasks are very doable.”

Many patients and family partners viewed the evaluation as an additional task. One member, MG, further explained, “PEIRS has nothing to do with my work in the research. It's not a reflection of my contribution to the project. It feels like just another task. It's an evaluation.” The PEIRS evaluations might seem to be burdensome to some partners. Some of them described the survey as “a chore,” “tedious,” and “a to-do task.”

### 3.2 The adaptations

#### 3.2.1 Questions in PEIRS

The PEIRS survey has 22 questions. Some members found some questions among the 22 questions to be similar. One patient partner, LJ, said, “It's repetitive. It's just tedious.” Our team member, KW, one of the PEIRS-22 interviewers, said, “I think at a certain point when I was going through the survey, I was thinking why I am asking the same questions again.” She further shared her concern, “There is a possibility that people may not even go through the [repeated or similar] question, because both the person asking and the person receiving may come to a consensus, ‘whenever the question is similar, just skip it.”' For example, the questions under the subthemes “Procedural Requirements” and “Contributions” regarding the use of time by our partners sound similar. Another set of identical questions are related to our partners' decision making in the project under the subthemes “Benefits” and “Procedural Requirements.”

Our patient partner MG raised the potential for conflicts in people's answers to similar questions: “If you answer A on the first one, and you answer C on the next similar question. Then, which answer is correct? So there is confusion and a danger [for conflict of answer] there.” Our project lead, JM, commented that the interviewers could take this opportunity to learn from and build on the previous answer to a similar question. For example, interviewers can explore further when there are discrepancies in the answers to the two questions.

#### 3.2.2 Adaptations for older adults living with dementia and family partners

The team reflected on strategies to encourage people to answer the PEIRS survey. One patient partner, LJ, questioned, “I don't know how it [PEIRS] could be made more interesting so that the questions are more amiable to answer.” Our patient partner, MG, appreciated the use of emojis adopted by our team for the PEIRS-22. He commented, “Using emojis makes it [PEIRS] a little bit fun to answer rather than having a series of questions, especially for people with a shorter focus and attention span.” He also suggested that using an online survey tool might help design a survey that is “easier and fun to answer.” Our patient partner LJ responded to MG's suggestion based on her perspective as a person living with dementia: “If we are looking for designs for people with dementia, using these online survey tools might be tricky for them to navigate and complete the survey.”

Our evaluation team lead, CB, commented on the adaptations of the PEIRS-22 in our project, “At the very beginning, the team had a difficult time designing the online survey. We separated the sessions so that there are only 3 to 7 questions per page, which doesn't feel and look so long for the respondents.” Our partners also liked another adaptation of adding a comment box to each question. MG said, “The comment boxes give a richer data collection because you cannot just say agree or neutral, but if you have a comment box and this adds a layer of information, I think that might be helpful.” Our family partner, AB, added, “It is really good to add the comment boxes. The intention is to allow for more personalized comments.”

### 3.3 The factors influencing its implementation as an evaluation tool

#### 3.3.1 The interviewer's identity

One of the factors discussed by the team regarding the implementation of the PEIRS-22 is the person conducting the PEIRS interview. Some members questioned whether the participants' answers would change due to the relationships between interviewees and interviewers. Our project lead, LH, stated, “I have assumptions and a lot of positivity. If I were the person to ask for feedback, people might tell me good things and try to be polite. However, we wanted to know what matters most to them so that we can improve. People may not tell me because they try to be polite.” Our evaluation team lead, CB, added the positive aspect of having arm-length interviewers to the project: “Being an outsider of the research project, the interviewer can be neutral and less likely to bring people to positive responses when asking questions. Interviewers could be more genuine, curious, and remain curious.” One member also raised an interesting hypothesis on whether the participants would answer differently if the survey interviewer was an older adult or of similar cultural background: “Having similar age and cultural background may open up more conversations for feedback during the evaluations.”

Some members, like AB and LJ, commented that their responses as participants would not alter based on whether they knew the person who did the interview. AB said, “I don't really think that it [who the interviewer is] would affect my responses. I generally have no problems being negative […] It is sort of giving my impression of things.”

#### 3.3.2 The confidentiality of responses and follow-ups

Our team conducted multiple rounds of the PEIRS-22 in an anonymous format with online digitalized surveys. Except for the partner who preferred using Zoom interviews for the evaluation, the comments from other partners shared on the digitalized version were confidential. The interviewers pointed out that the anonymity made it challenging to follow up with the participants, for example, on the progress of the team engagement performance and whether the team had any improvements with participants' feedback and addressed their concerns. When the team reflected on whether we should maintain anonymity for the evaluations of engagement in the research, the participants had diverse opinions. One family partner, AB, said, “I usually choose to have my name revealed. It doesn't matter to me whether it's anonymous or not. I will probably still say the same thing.” However, our patient partner, MG, was concerned about the impact on some respondents, even if they learned that their names would only be known to the interviewers. MG stated, “Revealing names to interviewers might impact the nature of feedback received… Participants may hesitate to respond freely if their identities are attached.”

Our project lead, LH, reflected, “In hindsight, we didn't have a discussion about anonymity during the evaluation planning. ‘Would you prefer anonymity?”' It is crucial for patients and family partners to grasp the significance of disclosing their identities, including options to remain anonymous or to be identified. They should be given the necessary information to decide for themselves whether to reveal their identities to interviewers and research teams. For instance, the research team could illustrate how the disclosure of names to interviewers could be beneficial for follow-up actions related to team performance.

Regarding follow-up evaluations, MG, drawing on his experience with dementia, underscored the potential memory problem of participants regarding their responses, advocating for a reminder about follow-up inquiries at the PEIRS evaluation's conclusion: “A prompt at the end of the evaluation should be included to inform participants of subsequent follow-ups,” he suggested. Our patient partner co-lead, JM, recommended offering follow-up options in the survey, such as “If we could follow up with you, please give us your preferred future contact.” Additionally, MG proposed a system to maintain confidentiality by assigning numbers to names, enabling follow-up without revealing identities: “Assign a unique number to each participant, which will be known only to the interviewer. This allows responses to be tracked while maintaining confidentiality.”

Our family partner, AB, emphasized the need to carefully consider the specific questions to follow up on: “We need to reflect on the important points that need further investigation and the ones that we are really focusing on. The evaluating team needs to take time to explore what we want to understand […] which questions we want or need to dig deeper.” For example, one evaluation found that patient partners were unclear about their tasks or roles. After some strategies were in place, the interviewers could follow up in the subsequent evaluation interview on whether the patient partner felt clearer about the project tasks to contribute and manage the tasks better. The interviewer, CB, noted that questions receiving a “neutral” response without additional comments should be examined more closely by the team for deeper insights.

#### 3.3.3 The timing and frequency of using the tool

Another factor for implementing the PEIRS evaluations is the timing of conducting the PEIRS survey, as our project had multiple rounds of evaluations. The project coordinator, JW, shared that more comments and suggestions were received at the beginning: “Personally, I think the very first one [PEIRS evaluation] is the most useful because there are more comments and suggestions. We made quite a lot of changes and improvements after the first one.” For example, some partners voiced out that there needed to be external sources of communication besides team meetings as there might be too much information in a meeting. The team thus moved some information in the email. After that, the team found a more innovative way than emailing, which was sharing information via monthly newsletters. The research team received an encouraging comment in the subsequent PEIR-22 evaluation: “Fabulous newsletter! Very engaging design and succinct messaging. As mentioned above, the newsletter really provided a great opportunity to get to know one another through photos and feature stories.” The evaluation lead, CB, also noticed a decrease in the number of comments shared in the later rounds of the PEIRS-22, “During the third or the fourth time [of evaluation], there were not as many comments in the comment boxes.” Our project lead, JM, also suggested external factors impacting the scoring of the PEIRS that might not be related to the research project, such as personal life events. The evaluation team lead, CB, echoed and shared that the third evaluation, in which the team got a lower average score, was done during a time of sustained stress during the COVID-19 pandemic. The availability of vaccines and the provincial restrictions might be potential external factors impacting patient and family partners during the third evaluation.

Some members commented on the relationships between questions and the time of evaluations. MG commented on the relevance of some questions to be asked at a certain time of the research progress: “At the beginning of the project, I thought the project was not completed yet, so why are we asking for a conclusion already on how we feel about the project?” One example regarding MG's comments is the question under the subtheme “Convenience” about the time allowed for completing his assigned tasks in the project. Our family partner LW also expressed that at the beginning of the project, she found it difficult to answer the questions regarding tasks, contributions, and workload when she was still exploring the project details and her role. One question LW mentioned regarding her contributions is under the subtheme “Procedural Requirements.” These might explain some blank answers received in the survey where the questions might not be applicable during evaluation. A family partner, AB, shared that having a PEIRS survey to be conducted right after a project meeting would be helpful. She said, “It [The experience] was much fresher in my mind. My answers [to the PEIRS survey] were more relevant to the project context.”

Although one family member, AB, appreciated that using the same set of standardized questions at different time points of the project could provide a basis for comparison over time, some members questioned the purpose of repeating the same questions for every evaluation. For instance, one member felt confused about the repeated questions: “There was a little bit of ‘Why are they asking it again?' It's repetitive. So maybe if you do it less often, we may not find it repetitive […] Say, maybe do it in the middle and the end.”

#### 3.3.4 The presentation of the evaluations

Our project's first round of the PEIRS evaluations were all one-to-one online interviews. After that, one member continued with online individual interviews, while most preferred the self-administered online survey. Our team reflected on the preferred evaluation format, whether it should be a facilitated interview or a self-administered survey, an individual or group interview, or in-person or Zoom meetings. Our patient partner, MG, emphasized the potential “side effects” of personal interviews:

“No personal interviews at all. Most people are polite. I don't want you [the interviewers] to feel offended because everybody works hard, and I don't give failing marks. If I do it at home or on paper, then it gets shown in the table, or it goes to the data. Who cares who I was talking to […] I would not recommend a personal interview if you know exactly what you want and an input that is not biased.”

Another member, LJ, shared her preference for completing an online self-administered survey on her own even though her answers would not be impacted by having an interviewer: “I would prefer to do an online or a paper one [survey] by myself. And you know, the interviewers, either way, if it was in person or Zoom, I don't have a problem saying how I feel, so that wouldn't be a problem for me.” Our project lead, LH, suggested the option of a group evaluation session: “Group sessions might help facilitate a better kind of conversation. So it's not as boring.” Our member MG responded to the suggestion of having group evaluations: “There might be a group dynamic. They [The participants] might be persuaded by other people [in the group] to say, ‘I agree'. Or sometimes when others say, ‘I don't like this,' ‘Yes, I don't like this either.”'

For the online option, our patient partner, MG, raised the concern of accessibility for the population our project team is engaging. He said, “We have to think about the older adults who are not conversant with technology.” Our project lead, LH, echoed and reiterated the importance of having options and flexibility: “People are not homogeneous. We all have different preferences. We need to offer people options and understand what meets people's needs.”

## 4 Discussion

There is an emerging trend to engage people with lived experience and people living with dementia in research (Miah et al., 2019; Williams et al., 2020; Vellani et al., 2023). However, there is a lack of evidence on using validated tools to evaluate the impact and process of public engagement and inclusion of people with lived experiences in dementia research (Miah et al., 2019). This reflection paper contributes to the field of dementia research by documenting how the PEIRS-22 tool can be used over multiple time points to evaluate team engagement in a dementia research project and by sharing critical team reflections on the key learnings in adopting the tool. Based on the key learnings, we will discuss (1) the need for adaptations and reflections, (2) insights for using evaluation tools with older adults living with dementia, and (3) the opportunity to build a community of learning in the field to improve engagement in dementia research. We offer practical tips and implications for future research.

One message that stood out from the team reflections is that researchers need to acknowledge that evaluation tools such as the PEIRS survey are not “one size fits all.” As suggested by Mann and Hung (2019, p. 587) in the “ASK ME” framework of tips for engaging a person with dementia in research, one practical tip is “to support the person to do the best.”: “It is useful to take the time to get to know the person […] Support the person to maximize contribution and avoid exploitation. See the person with an appreciative lens helps to focus on strengths and possibilities.” Despite using the same tool, researchers may need to adapt and tailor the use of the evaluation tool to support and maximize responses from the specific group of people with lived experiences in the research team. There should be flexibility and a shared informed decision-making process early in the project on how the team will work with the engagement evaluation procedures, e.g., the format and time of the evaluations and who should be performing the evaluations. These factors may have potential positive or negative influences on the evaluation and the partners' experiences in the evaluation process. Providing options and having co-developed strategies can facilitate the evaluations. Ongoing critical reflections are needed to examine the use of evaluation tools among the team. Ensuring a shared consensus and clear understanding of how and whether the evaluation tool helps the project team improve is essential. Otherwise, the initial intention behind evaluating to improve engagement may, in turn, become “a burdensome chore” to people with lived experiences. It may lead to negative engagement experiences for these individuals due to a complicated evaluation process.

For the population that our team is working with, which includes people living with dementia and older adults, several aspects need to be considered based on the critical reflections. The evaluation timing should be carefully considered when working with this population. For example, evaluations can be done right after completing specific project tasks or milestones, e.g., manuscript writing, to allow people to provide feedback regarding the engagement process to provide an “at present” feeling. The considerations of the timing of performing the evaluations echoed a recent reflection on a study using a loneliness scale for people living with dementia (Wong et al., 2022). Participants in the study tended to respond to how they felt during the interview rather than recalling their past experiences or feelings. Besides finding suitable evaluation timing, having a dementia-friendly survey can help facilitate the evaluations. The research team can refer to online resources on developing dementia-friendly surveys (Alzheimer's Society, n.d.) or co-create in-print and online surveys with people with lived experiences. Tailoring the survey to be more dementia-friendly and fun will offer individuals easier navigation of the evaluation tool and a more enjoyable evaluation experience.

This reflection underscores the significance of collective learning about the evaluation of research engagement of people with lived experiences within the field. Our team had little evidence to guide our engagement evaluations with people living with dementia at the beginning of the project. Continuous dialogues and discussions in the field are crucial to exploring what works and what does not work in the evaluation processes in various projects, how different teams adopt and adapt evaluation tools, how researchers can improve evaluation tools, what meaningful engagement means to different populations, and how the engagement experiences of patient and family partners can be better supported. The sharing of opportunities and challenges of diverse research teams allows improvements for future teams to adopt evaluation tools and contributes to the continuous development of evaluation methods to support patient and public engagement. Creating a learning community for evaluating research engagement resonates with that of a “learning health system” suggested by L'Espérance et al. (2021), which could help build capacity for implementing patient and public engagement and evaluations across the research community.

This reflection embraces assumptions, feelings and experiences of our patient and family partners and team members, which reminds our team and scholars that engagement evaluation should not only focus on the “performance” or “score.” It may be more important to understand better and enhance how patients and family partners “feel,” which is beyond the scores research teams obtain from validated measures.

### 4.1 Practical tips

Based on our key learnings, we offer the following six practical tips, “ENGAGE” (see Table 3). These tips can inform future research projects, particularly studies in the dementia field, to adopt the PEIRS-22 or other evaluation tools to enhance patient engagement experiences.

**Table 3:** Description of six practical tips “ENGAGE”.

**E**	It is an enjoyable and fun process	The evaluation enhances patient engagement experiences but not adds an extra burden on patient and family partners. It should also be beneficial and enjoyable. Adopting elements like emojis can make the evaluation process fun and more comfortable.
**N**	Never impose	The team needs to acknowledge that people are heterogeneous and have different preferences. The evaluation methods and process should be flexible and a shared decision among the team. Nothing should be imposed on team members.
**G**	Get prepared early	The preparation should involve the whole team from the beginning of the project, e.g., the aim and process of the evaluation, anonymity, the evaluation tool and the timing of evaluations. Gentle reminders from time to time on the important evaluation components (why and how) help keep people living with dementia informed and prepared.
**A**	Adapt to the team's needs	Different research teams may involve diverse groups of people with lived experiences who have specific needs, e.g., language barriers due to cultural backgrounds, education backgrounds and cognitive and physical challenges. For example, in our team, it was easier and fun for people living with dementia and older adults to answer the rating scale with emojis.
**G**	Give people options	Giving evaluation respondents options shows respect by the research teams. Individuals in the team can enjoy autonomy in deciding for themselves their preferences, e.g., being anonymous and choosing the evaluation format. This enables a person-centered approach in the evaluation process.
**E**	Evaluate and reflect	Teamwork and self-reflection on the engagement evaluation process are necessary to ensure that the use of the evaluation tool is meaningful and helpful in enhancing engagement experiences. Without reflection, the evaluation process of engagement experiences may become a “routine” and “wasted” task for the project team.

### 4.2 Implications for future research

In our team reflections, it was noted that some questions seemed repetitive when the PEIRS-22 evaluations were performed multiple times in a project. Future studies can explore whether there is a need to modify or omit some questions when the survey is repeated at different time points in a project. Another comment is on the impact of interviewers during the evaluation process. Future research can explore the impact of different interviewers on the evaluation results, e.g., whether it would be more beneficial to have an outsider than an insider of the project team as the evaluation interviewer.

Regarding the development and validation of evaluation tools, the PEIRS-22 has not been validated with people living with dementia. Future research developing evaluation tools for this population can engage people living with dementia and their care partners in the tool development and validation process. Including people with lived experiences can ensure the tool developed is relevant, meaningful, and accessible to the targeted population. Furthermore, our research team's patient partner co-lead is living with an early stage of Alzheimer's disease. Future research can explore the use of evaluation tools and strategies with research team members living with different types of dementia and individuals with diverse backgrounds.

## 5 Conclusions

Given the emerging trend of including people with lived experiences in dementia research, there is a need to continuously evaluate the engagement experiences of patient and family partners and the engagement strategies adopted by the research team. With a lack of studies documenting the use of evaluation tools and evaluation processes with people living with dementia, the key learnings from using the PEIRS-22 in a Canadian patient-oriented research study offer pragmatic insights and tips for future research teams on using engagement evaluation tools. Researchers can co-plan different aspects of the evaluation process with patient and family partners. Having ongoing critical reflections is key to more effective use of evaluation tools to enhance engagement. When more research teams share the challenges and opportunities regarding engagement evaluations, a community of practice and learning can be built to support one another on the journey of public and patient engagement in dementia research.

## Data availability statement

The raw data supporting the conclusions of this article will be made available by the authors, without undue reservation.

## Ethics statement

The studies involving humans were approved by UBC Behavioural Research Ethics Board. The studies were conducted in accordance with the local legislation and institutional requirements. The participants provided their written informed consent to participate in this study. Written informed consent was obtained from the individual(s) for the publication of any potentially identifiable images or data included in this article. Participants provided verbal consent and were offered the option to be identified by their actual names or pseudonyms in the dissemination of findings. Each participant has reviewed and approved the contents of this article.

## Author contributions

JW: Writing – review & editing, Writing – original draft. LH: Supervision, Funding acquisition, Conceptualization, Writing – review & editing. CB: Writing – review & editing, Writing – original draft. KW: Writing – original draft, Writing – review & editing. AB: Writing – review & editing. JM: Writing – review & editing. LW: Writing – review & editing. LJ: Writing – review & editing. MG: Writing – review & editing.
