# Corrigendum: A critical reflection on using the Patient Engagement In Research Scale (PEIRS) to evaluate patient and family partners' engagement in dementia research

**DOI:** 10.3389/frdem.2024.1490895

**Published:** 2024-09-18

**Authors:** Joey Wong, Lillian Hung, Cates Bayabay, Karen Lok Yi Wong, Annette Berndt, Jim Mann, Lily Wong, Lynn Jackson, Mario Gregorio

**Affiliations:** ^1^School of Nursing, University of British Columbia, Vancouver, BC, Canada; ^2^UBC IDEA Lab, School of Nursing, University of British Columbia, Vancouver, BC, Canada; ^3^School of Social Work, University of British Columbia, Vancouver, BC, Canada

**Keywords:** patient and public involvement, aging, dementia, older adults, technology, evaluation

In the published article, there was an error in Figure 1 as published. Figure 1 has now been removed entirely in order to unpublish this incorrect material.

In the published article, there was an error including the scale items. A correction has been made to **Section 3.2**, *Subsection 3.2.1*, Paragraph 1. This sentence previously stated:

“For example, the question under the subtheme “Procedural Requirements”—“The project was worth the time I spent on it” and the subtheme “Contributions”—“My contributions were a good use of my time” sounds similar. Another set of identical questions is “I made an impact on the decisions in the project” under the subtheme “Benefits” and “I participated in making decisions about the project” under the subtheme “Procedural Requirements.””

The corrected sentence appears below:

“For example, the questions under the subthemes “Procedural Requirements” and “Contributions” regarding the use of time by our partners sound similar. Another set of identical questions are related to our partners' decision making in the project under the subthemes “Benefits” and “Procedural Requirements.””

In the published article, there was an error including the scale item. A correction has been made to **Section 3.3**, *Sub-section 3.3.3*, Paragraph 2. This sentence previously stated:

“One example regarding MG's comments is the question under the subtheme “Convenience”—“Throughout the project, I had sufficient time to complete my tasks for the project.””

The corrected sentence appears below:

“One example regarding MG's comments is the question under the subtheme “Convenience” about the time allowed for completing his assigned tasks in the project.”

In the published article, there was an error including the scale item. A correction has been made to **Section 3.3**, *Sub-section 3.3.3*, Paragraph 2. This sentence previously stated:

“For example, the question under “Procedural Requirements”—“In general, I had sufficient opportunities to contribute to the project.””

The corrected sentence appears below:

“One question LW mentioned regarding her contributions is under the subtheme “Procedural Requirements.””

The authors apologize for these errors and state that they do not change the scientific conclusions of the article in any way. The original article has been updated.

